# Cation ATPase (ATP4) Orthologue Replacement in the Malaria Parasite Plasmodium knowlesi Reveals Species-Specific Responses to ATP4-Targeting Drugs

**DOI:** 10.1128/mbio.01178-22

**Published:** 2022-10-03

**Authors:** Franziska Mohring, Donelly A. van Schalkwyk, Ryan C. Henrici, Benjamin Blasco, Didier Leroy, Colin J. Sutherland, Robert W. Moon

**Affiliations:** a Department of Infection Biology, Faculty of Infectious and Tropical Diseases, London School of Hygiene and Tropical Medicine, London, United Kingdom; b Center for Global Health, University of Pennsylvania School of Medicine, Philadelphia, Pennsylvania, USA; c Medicines for Malaria Venture, Geneva, Switzerland; d UK Health Security Agency Malaria Reference Laboratory, London School of Hygiene and Tropical Medicine, London, United Kingdom; National Institute of Allergy and Infectious Diseases

**Keywords:** ATP4, CRISPR-Cas9, malaria, *Plasmodium falciparum*, *Plasmodium knowlesi*, *Plasmodium ovale*, *Plasmodium vivax*, antimalarial agents

## Abstract

Several unrelated classes of antimalarial compounds developed against Plasmodium falciparum target a parasite-specific P-type ATP-dependent Na^+^ pump, PfATP4. We have previously shown that other malaria parasite species infecting humans are less susceptible to these compounds. Here, we generated a series of transgenic Plasmodium knowlesi orthologue replacement (OR) lines in which the endogenous *pkatp4* locus was replaced by a recodonized *P. knowlesi* atp4 (*pkatp4*) coding region or the orthologous coding region from P. falciparum, Plasmodium malariae, Plasmodium ovale subsp. *curtisi*, or Plasmodium vivax. Each OR transgenic line displayed a similar growth pattern to the parental P. knowlesi line. We found significant orthologue-specific differences in parasite susceptibility to three chemically unrelated ATP4 inhibitors, but not to comparator drugs, among the P. knowlesi OR lines. The PfATP4^OR^ transgenic line of P. knowlesi was significantly more susceptible than our control PkATP4^OR^ line to three ATP4 inhibitors: cipargamin, PA21A092, and SJ733. The PvATP4^OR^ and PmATP4^OR^ lines were similarly susceptible to the control PkATP4^OR^ line, but the PocATP4^OR^ line was significantly less susceptible to all ATP4 inhibitors than the PkATP4^OR^ line. Cipargamin-induced inhibition of Na^+^ efflux was also significantly greater with the P. falciparum orthologue of ATP4. This confirms that species-specific susceptibility differences previously observed in *ex vivo* studies of human isolates are partly or wholly enshrined in the primary amino acid sequences of the respective ATP4 orthologues and highlights the need to monitor efficacy of investigational malaria drugs against multiple species. P. knowlesi is now established as an important *in vitro* model for studying drug susceptibility in non-falciparum malaria parasites.

## INTRODUCTION

Malaria continues to exert a substantial cost on human health, with 627,000 deaths from this disease estimated for 2020, despite encouraging progress toward elimination in some settings (1). Plasmodium knowlesi, a zoonotic malaria, accounted for almost all malaria cases reported in Malaysia in 2019 (3,212 P. knowlesi infections), with no indigenous cases of human malaria reported in that year (2). Importantly, P. knowlesi has been shown to exhibit a case fatality rate of 2.5/1,000 cases and is the most common cause of malaria deaths in Malaysia (3).

WHO guidelines recommend artemisinin combination therapy (ACT) for the treatment of uncomplicated malaria (4). Partial resistance to artemisinin derivatives and resistance to ACT partner drugs such as piperaquine has arisen in the Greater Mekong subregion (GMS) (5–7) and is now also being reported in Africa, where the burden of malaria is greatest (8, 9). If not checked, falling susceptibility to ACT in Africa would risk an increase in mortality, as was seen during the rise of chloroquine resistance in the late 20th century (10). New drug development is critical to circumvent this eventuality and maintain progress toward malaria elimination.

All drugs in use today for malaria treatment were identified before the turn of the century. However, with the development of high-throughput *in vitro* screens against live parasites, the last 2 decades have witnessed a revolution in malaria drug discovery, identifying numerous chemical scaffolds for drug development and several novel mechanisms of drug action (11–14). A particular success has been the development of the spiroindolone cipargamin (KAE609), which potently inhibits the growth of multidrug-resistant Plasmodium falciparum lines *in vitro* and Plasmodium vivax and P. falciparum isolates *ex vivo*, with half-maximal effective concentration (EC_50_) values of <10 nM (15). Cipargamin was the first compound with a novel mechanism of action to enter human trials in 20 years (16). Spiroindolones, including cipargamin, disrupt Na^+^ homeostasis in malaria parasites, and spiroindolone-resistant lines harbor mutations in the gene encoding PfATP4, a *P. falciparum* P-type Na^+^ ATPase (17). Two unrelated antimalarial compounds, SJ733 and PA21A092, have also been shown to disrupt Na^+^ homeostasis in the malaria parasite, and mutations in PfATP4 are also implicated in resistance to these nonspiroindolone molecules (18, 19).

A collection of 525 structurally diverse compounds that target malaria parasites, assembled by the Medicines for Malaria Venture (MMV), has been made available to the scientific community for evaluation. Thirty-nine of these structurally diverse antimalarial chemotypes have been shown to disrupt Na^+^ homeostasis in the malaria parasite (20, 21). This convergence around a single cellular process (regulation of intracellular sodium concentration through ATP4) confirms this as a critical drug target and a potential Achilles heel for the parasite.

ATP4 is an essential protein (22) that is localized to the parasite plasma membrane (15). Inhibition of ATP4 causes an increase in the parasite’s intracellular Na^+^ concentration ([Na^+^]_i_) and a concomitant increase in cytosolic pH (17). While ATP4 was initially annotated as a Ca^2+^ transporter (23), current evidence supports ATP4 functioning as an Na^+^ efflux ATPase that exports Na^+^ while importing H^+^ (17).

While multiple ATP4 inhibitors have been identified as highly potent against P. falciparum *in vitro* and *ex vivo*, evidence of potency against other malaria species has been mixed. Cipargamin and the pyrazoleamide PA21A092 were shown to be effective against both P. falciparum and P. vivax parasites using *ex vivo* screens (15, 19), although EC_50_ data generated in this way is difficult to compare cross-species due to the large variance in the estimates for P. vivax. However, the dihydroisoquinolone SJ733 was estimated to be 10-fold less potent against Plasmodium berghei and two other murine malaria species than against P. falciparum using *ex vivo* tests (18). We have previously compared the activities of ATP4 inhibitors against P. falciparum (3D7 clone) and the zoonotic P. knowlesi (A1-H.1 clone) under identical *in vitro* conditions. P. knowlesi was found to be approximately 6-fold less susceptible to cipargamin, SJ733, and PA21A092 than P. falciparum (24). Furthermore, we confirmed that two other human-infecting malaria parasites (Plasmodium malariae and Plasmodium ovale spp.) are 5- to 7-fold less susceptible to cipargamin than P. falciparum when tested under identical *ex vivo* conditions (25). This suggests that there may be important species differences in susceptibility to these ATP4 inhibitors, with P. falciparum being the most susceptible of the human malaria species so far tested.

In an effort to explain the differences in susceptibility of SJ733 between the murine malaria species and P. falciparum, Jimenez-Diaz et al. performed an ATP4 orthologue replacement (ATP4^OR^) in P. berghei (18). The authors found that when the P. berghei *atp4* (*pbatp4*) gene was replaced with *pfatp4*, the resulting parasites were more susceptible to SJ733 in their *ex vivo* screen (18). This suggests sequence variation between the *pbatp4* and *pfatp4* genes is responsible for the species differences in susceptibility to SJ733. It remains unknown whether the differences we have observed in susceptibility to multiple ATP4 inhibitors among the human-infecting malaria species are similarly determined by sequence variations among the different orthologue genes.

To further understand and characterize observed differences in susceptibility to ATP4 inhibitors among human-infecting malaria species, we applied an orthologue replacement (OR) approach in our now well-established P. knowlesi *in vitro* drug susceptibility model. We replaced the endogenous *pkatp4* gene with codon-harmonized *atp4* genes from each of the other human-infecting malaria species: P. falciparum (*pfatp4*), P. vivax (*pvatp4*), *P. malariae* (*pmatp4*), *P. ovale* subsp. *curtisi* (*pocatp4*), and P. knowlesi (*pkatp4*). We then compared the P. knowlesi transfectant lines to assess growth rates, drug susceptibility, and potential changes in [Na^+^]_i_ and pH. Finally, we used molecular modeling to explore potential mechanistic explanations for the species differences identified among our transfectant lines.

## RESULTS

### Orthologue replacement of ATP4 in P. knowlesi does not impact parasite growth.

We utilized our *in vitro* P. knowlesi model to study possible species differences in susceptibility to ATP4 inhibitors. We have previously shown the value of adapting our P. knowlesi model to evaluate P. vivax Duffy binding protein vaccine candidates (26, 27). As with *P*. *malariae* and *P*. *ovale* spp., P. vivax lacks a long-term *in vitro* culture system (28).

**FIG 1:**
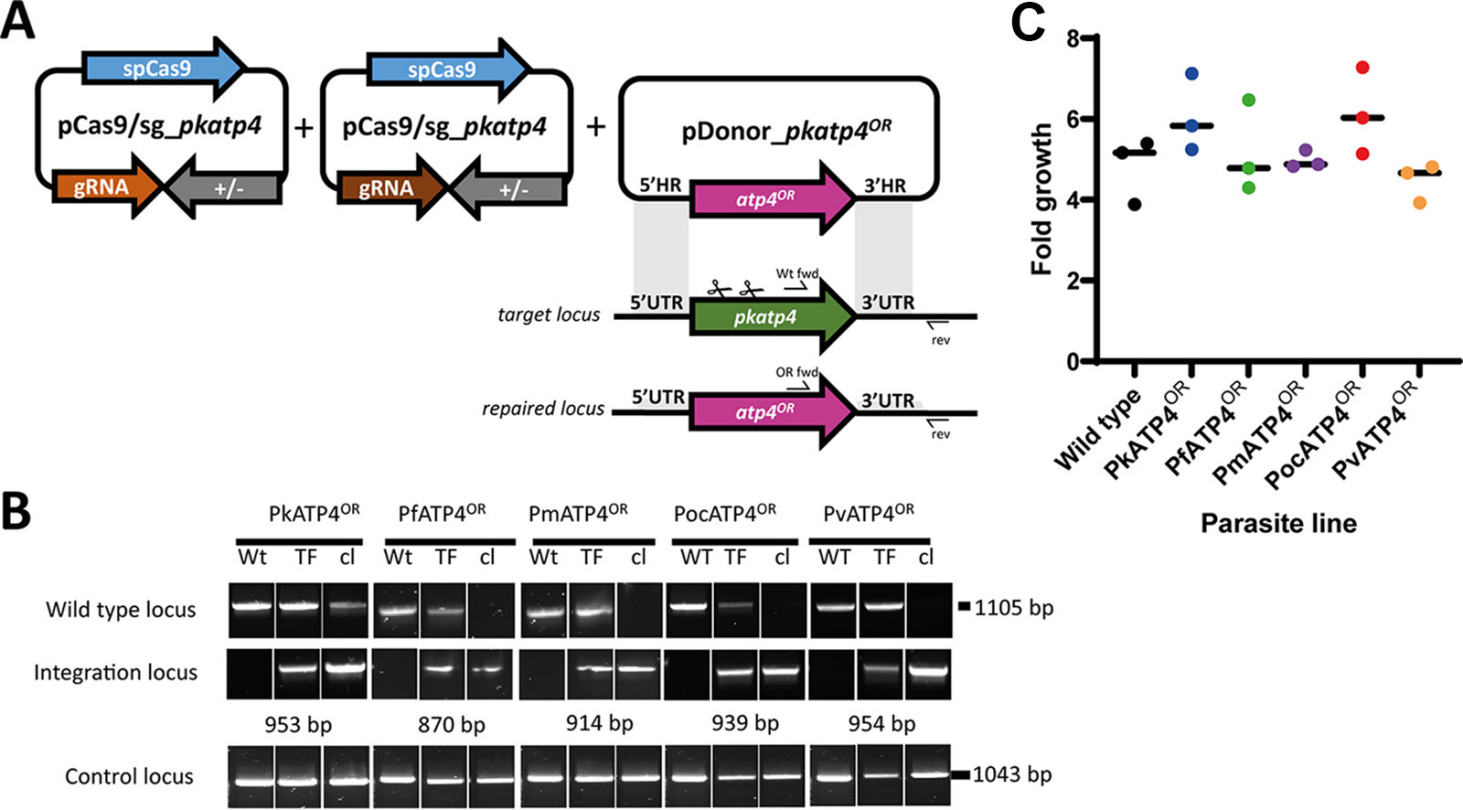
Orthologue replacement (OR) of Plasmodium knowlesi ATP4. (A) Schematic of CRISPR-Cas9 genome editing strategy. Integration of *atp4* orthologues into the target *pkatp4* locus was via homologous recombination. Arrows indicate oligonucleotide positions for diagnostic PCRs. (B) Parasites transfected (TF) with pCas9/sg_ATP4 and pDonor_atp4^OR^ plasmids were analyzed with diagnostic PCRs. Shown are results from PCRs detecting the wild-type locus (i.e., from the parental A1-H.1 clone) (Wt fwd + rev) and integration locus (Int fwd + rev) as well as a control PCR targeting an unrelated locus (PkMTIP fwd + PkMTIP rev). The WT locus band for the PkATP4^OR^ clonal line was confirmed as PkATP4^OR^ by amplicon sequencing. (C) Graph showing fold multiplication of WT, PkATP4^OR^, PfATP4^OR^, PmATP4^OR^, PocATP4^OR^, or PvATP4^OR^ parasites in erythrocytes over one intraerythrocytic growth cycle (27 h). Assays were carried out in technical duplicates in Duffy-positive erythrocytes with three independent biological replicates. A one-way ANOVA revealed there was no statistically significant difference in growth rates between at least two groups: *F*(5, 12) = [1.966] and *P* = 0.16.

Transgenic P. knowlesi A1-H.1 parasites were generated using the CRISPR-Cas9 genome editing approach described by Mohring et al. (26). The entire locus of *pkatp4* was replaced with either a recodonized version (PkATP4^OR^), which encodes an identical amino acid sequence with altered codon usage at the nucleotide level, or recodonized ATP4 orthologues of P. vivax, P. falciparum, *P. malariae*, and *P. ovale* subsp. *curtisi* (PlasmoDB.org) (29–33) (Fig. 1A). Following cotransfection of each plasmid bearing a synthetic ATP4 orthologue together with the Cas9-containing companion plasmid, successful integration was readily achieved for all ATP4 orthologues and clonal lines established for each by limiting dilution cloning (Fig. 1B). ATP4 is essential for malaria parasite survival (22); thus, the successful replacement of *pkatp4* indicates that the P. falciparum, P. vivax, *P. malariae*, and *P. ovale* subsp. *curtisi* orthologues can complement its cellular function. Furthermore, growth assays showed no significant differences between the parental P. knowlesi A1-H.1 line and the five transgenic lines (Fig. 1C).

### ATP4 orthologue replacement in P. knowlesi confers species differences in susceptibility to ATP4 inhibitors but not to comparator drugs.

**FIG 2:**
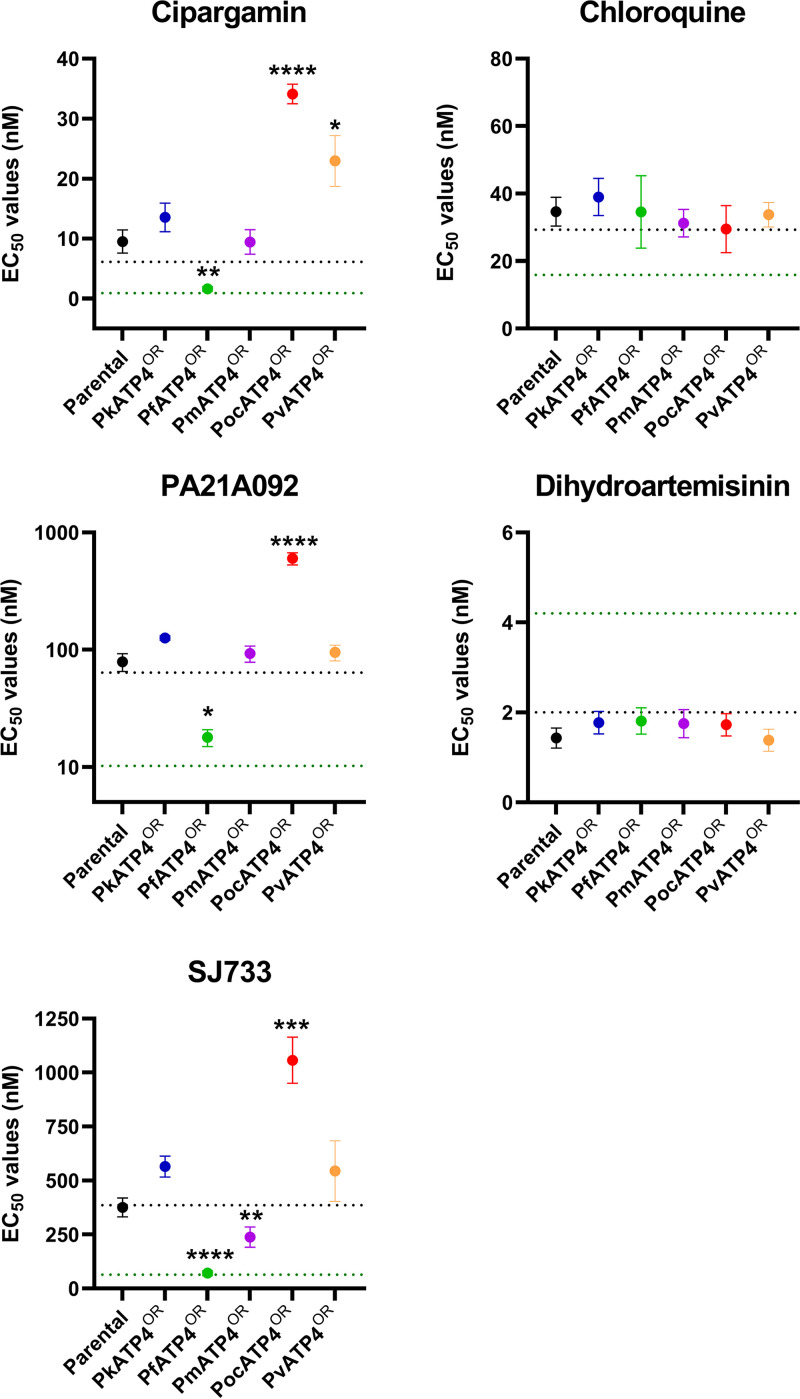
Drug susceptibility of orthologue replacement lines. Parental or orthologue replacement (OR) lines were exposed to serial dilutions of ATP4 inhibitors (cipargamin, PA21A092, and SJ733) or control drugs (chloroquine and dihydroartemisinin) for one complete parasite life cycle (27 h). Parasite viability was measured using the SYBR green I fluorescence method. Data show the mean EC_50_ values (nanomolar concentration) from at least three experiments, with some including up to eight repeats. Error bars represent the standard error of the mean (SEM). Stars indicate parasite lines that were significantly different (*P* < 0.05) from the PkATP4^OR^ line (blue circles). For comparison also, using dotted lines, we plot where our previously published drug susceptibility data lie for the PkA1.H-1 (black lines) and Pf3D7 (green lines) parasite lines. These published data (24, 34) were from experiments performed under conditions identical (i.e., one life cycle exposure) to those used in the current study on the orthologue replacement lines. Note that the graph for PA21A092 is plotted using a log scale for clarity. All other graphs plot linear scales. *, *P* ≤ 0.05; **, *P* ≤ 0.01; ***, *P* ≤ 0.001; ****, *P* ≤ 0.0001.

To investigate the susceptibility of the different *Plasmodium* ATP4 orthologues to ATP4 inhibitors in the heterologous P. knowlesi cell, we conducted *in vitro* growth inhibition assays. Parasite lines, set to the same starting parasitemia and hematocrit, were exposed to serial dilutions of cipargamin, PA21A092, and SJ733 for one complete parasite life cycle (27 h). We also exposed each orthologue replacement line (denoted here by the superscript “OR”) to chloroquine and dihydroartemisinin, which exert their antimalarial effects independently of ATP4, as comparators. As expected, we did not observe significant differences in chloroquine or dihydroartemisinin susceptibility among the parental and orthologue replacement lines, nor were parental P. knowlesi parasites different in susceptibility to these drugs than the PkATP4^OR^ line, suggesting that the orthologue replacement process itself had not caused a general perturbation of drug responses (Fig. 2 and Table 1).

**TABLE 1:** Drug susceptibility of the parental P. knowlesi (A1-H.1 clone) and orthologue replacement lines to ATP4 inhibitors (cipargamin, PA21A092, and SJ733) and control drugs (chloroquine and dihydroartemisinin)^*a*^

Parasite line	EC_50_, nM (*P* value)				
	Cipargamin	PA21A092	SJ733	Chloroquine	Dihydroartemisinin
Parental	9.51 ± 1.9 (0.5878)	78.8 ± 13.6 (0.7185)	375 ± 44 (0.2104)	34.6 ± 4.3 (0.9899)	1.43 ± 0.22 (0.9238)
PkATP4^OR^^*b*^	13.5 ± 2.4 (NA)^*b*^	126 ± 5.5 (NA)	565 ± 48 (NA)	39.0 ± 5.5 (NA)	1.77 ± 0.25 (NA)
PfATP4^OR^	1.62 ± 0.37 (0.0019)	17.8 ± 2.9 (0.0317)	71 ± 11 (<0.0001)	34.6 ± 10.8 (0.9924)	1.81 ± 0.29 (0.9999)
PmATP4^OR^	9.44 ± 2.0 (0.5698)	92.8 ± 14.7 (0.9072)	238 ± 47 (0.0069)	31.2 ± 4.1 (0.8382)	1.75 ± 0.31 (0.9999)
PocATP4^OR^	34.1 ± 1.6 (<0.0001)	599 ± 70 (<0.0001)	1057 ± 107 (0.0001)	29.5 ± 7.0 (0.8222)	1.73 ± 0.25 (0.9999)
PvATP4^OR^	22.9 ± 4.2 (0.0172)	94.8 ± 14.6 (0.9433)	544 ± 140 (0.9997)	33.7 ± 3.6 (0.9845)	1.39 ± 0.24 (0.8725)
PkA1-H1^*c*^	6.1 ± 0.5 (0.0647)	63.8 ± 7.6 (0.3618)	386 ± 34 (0.2222)	29.3 ± 4.7 (0.6665)	2.03 ± 0.25 (0.9824)
Pf3D7^*c*^	0.89 ± 0.08 (0.0006)	10.2 ± 1.4 (0.0139)	64.3 ± 4.3 (<0.0001)	15.9 ± 3.0 (0.0222)	4.16 ± 0.52 (<0.0001)

a Parasites were exposed to drugs for one complete *in vitro* life cycle (27 h), and viability was measured using the SYBR green I method. Drug assays were run in technical duplicates on at least three biological replicates (up to eight times). Values are the mean ± SEM. *P* values were determined from *post hoc* analysis comparing EC_50_ data from all species to PkATP4^OR^ data using Dunnett’s test.

b NA, not applicable (as this is the comparator line).

c Data published previously on the P. knowlesi A1-H.1 line and on the P. falciparum 3D7 line in references 24 and 34 tested under identical conditions to the other lines tested here.

Among the ATP4 inhibitors screened, cipargamin was the most potent against all parasite lines, followed by PA21A092, while SJ733 was the least potent (Table 1). The PfATP4^OR^ transgenic line was the most susceptible to all ATP4 inhibitors tested (Fig. 2 and Table 1). For all ATP4 inhibitors, the PfATP4^OR^ line was significantly more susceptible than the control PkATP4^OR^ line (7- to 8-fold change) (see Table S1 in the supplemental material), which was in turn similar in susceptibility to PvATP4^OR^ and PmATP4^OR^ (Fig. 2, Table 1, and Table S1). Conversely, the PocATP4^OR^ line was significantly less susceptible to all ATP4 inhibitors than the control PkATP4 line (*P* < 0.0001) (Table 1 and Fig. 2). Notably, the PocATP4^OR^ line exhibited EC_50_ values 21, 33.7, and 14.9 times higher than those for the PfATP4^OR^ line for cipargamin, PA21A092, and SJ733, respectively (Table S1).

10.1128/mbio.01178-22.3 TABLE S1 Fold difference in EC_50_ values among the parental and orthologue replacement lines. Download Table S1, DOCX file, 0.01 MB. Copyright © 2022 Mohring et al. 2022 Mohring et al. https://creativecommons.org/licenses/by/4.0/ This content is distributed under the terms of the Creative Commons Attribution 4.0 International license.

For comparison, we include also previous data that we published for our P. knowlesi A1-H.1 line and P. falciparum 3D7 line in Table 1 (24, 34). As expected, the P. knowlesi EC_50_ estimates reported by us previously are similar to those derived here for the parental P. knowlesi line, this being the same parasite line (Table 1). Importantly, the susceptibility of the PfATP4^OR^ line to the three ATP4 inhibitors is similar to the susceptibility of the P. falciparum 3D7 line (24). However, the PfATP4^OR^ line is not similar in susceptibility to chloroquine or dihydroartemisinin compared to the P. falciparum line (Table 1). For these two comparator drugs, the susceptibility phenotype of the PfATP4^OR^ line is similar to that of the parental P. knowlesi line.

### Effect of orthologue replacement on intracellular sodium ([Na^+^]_i_).

**FIG 3:**
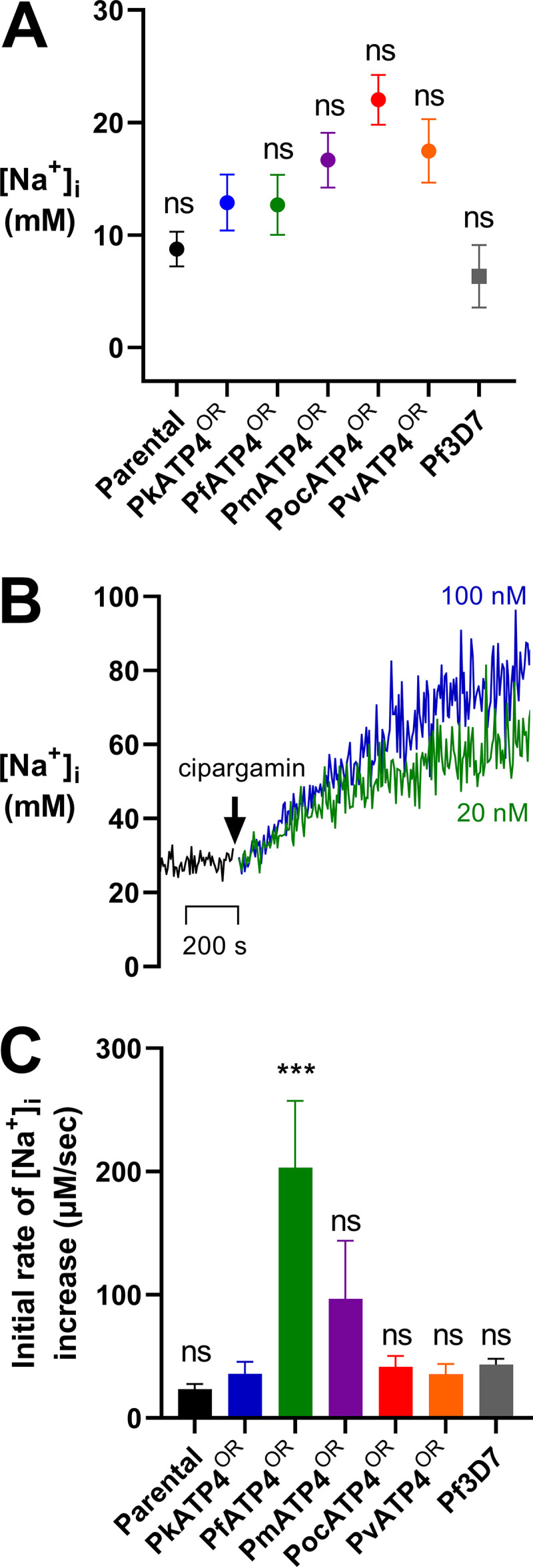
Resting intracellular sodium concentration ([Na^+^]_i_) of the orthologue replacement lines and the concentration-dependent increase in [Na^+^]_i_ after addition of cipargamin. Trophozoite-stage parasites were loaded with the Na^+^-sensitive fluorophore SBFI, and the fluorescence of the suspensions was measured in a spectrophotometer. (A) Measurements of resting [Na^+^]_i_ show values of between 6.34 mM for the P. falciparum 3D7 line (gray square) and 22.03 mM for the PocATP4^OR^ line (red circle). However, there was no significant difference for any of the lines compared to the PfATP4^OR^ line (green circle; *P* > 0.0681). Data show the mean ± SEM. (B) Addition of cipargamin resulted in an immediate dose-dependent increase in the resting [Na^+^]_i_ inside all parasite lines. Shown here are representative traces from a single experiment demonstrating the impact of adding 20 nM cipargamin (green trace) or 100 nM cipargamin (blue trace) on the resting [Na^+^]_i_ of the PocATP4^OR^ line. (C) Addition of 100 nM cipargamin to the parasite lines results in an increase in the [Na^+^]_i_ from steady state. We determined the initial rate of increase in the [Na^+^]_i_ by fitting a linear regression curve to the [Na^+^]_i_ increase data across that 10-min exposure. The slope of that curve provides the rate of increase (in micromolar concentration per second). Experiments for resting [Na^+^] were performed in technical duplicates for at least three biological repeats, but some were performed to eight times. Experiments for the initial rate of increase in [Na^+^] were performed in technical duplicates for at least three biological repeats, but some were performed up to five times. ***, *P* ≤ 0.001; ns, non significant (*P* > 0.05).

Previous research by Spillman et al. used the sodium-sensitive fluorescent dye SBFI to measure the resting intracellular sodium concentration ([Na^+^]_i_) of P. falciparum 3D7 parasites and the impact of adding ATP4 inhibitors on the resting [Na^+^]_i_ (17). They demonstrated that P. falciparum maintains a resting [Na^+^]_i_ of around 11 mM, and the addition of ATP4 inhibitors caused a dose-dependent increase in [Na^+^]_i_ (17). We observe average resting [Na^+^]_i_ values for our orthologue replacement lines of between 12.7 mM (PfATP4^OR^ line) to 22.0 mM (PocATP4^OR^ line) (Fig. 3A and Table 2). These resting [Na^+^]_i_ values of the transgenic lines were all slightly higher than the parental P. knowlesi line (8.75 mM) and the P. falciparum 3D7 line (6.34 mM) (Fig. 3), but these differences were not statistically significant compared to the PkATP4^OR^ line (*P* ≥ 0.0765) (Table 2).

**TABLE 2:** Comparison of the resting intracellular sodium concentration ([Na^+^]_i_) and the rate of increase and maximum increase in the [Na^+^]_i_ after a 10-min exposure to 100 nM cipargamin in the P. knowlesi A1-H.1 parental and orthologue replacement lines and the P. falciparum 3D7 line^*a*^

Parasite line	Resting [Na^+^]_i_, mM (*P* value)	Rate of increase in [Na^+^]_i_ 10 min after cipargamin exposure, μM/s (*P* value)	Maximum increase in [Na^+^]_i_ 10 min after cipargamin exposure, mM (*P* value)
Parental	8.75 ± 1.54 (0.7587)	23.3 ± 4.26 (0.9952)	17.9 ± 3.23 (0.9974)
PkATP4^OR^^*b*^	12.9 ± 2.48 (NA)^*b*^	35.8 ± 9.98 (NA)	26.2 ± 8.08 (NA)
PfATP4^OR^	12.7 ± 2.67 (>0.9999)	203.2 ± 54.1 (0.0002)	138.6 ± 34.1 (0.0007)
PmATP4^OR^	16.7 ± 2.43 (0.8913)	35.7 ± 8.07 (0.9999)	27.5 ± 6.59 (0.9999)
PocATP4^OR^	22.0 ± 2.21 (0.0765)	41.5 ± 8.85 (0.9997)	35.4 ± 7.97 (0.9962)
PvATP4^OR^	17.5 ± 2.81 (0.5835)	96.6 ± 47.3 (0.2618)	81.1 ± 39.9 (0.1312)
Pf3D7	6.34 ± 2.78 (0.3963)	43.5 ± 4.84 (0.9996)	33.3 ± 4.41 (0.9978)

a Resting [Na^+^]i, rate of increase, and maximum increase data were measured in SBFI-loaded saponin-isolated late-stage trophozoites suspended in physiological saline. All data show the mean ± SEM. Experiments for resting [Na^+^]_i_ were performed in duplicate on at least three occasions, but some were performed up to eight times. Experiments for rate of increase in [Na^+^]_i_ and maximum increase in [Na^+^]_i_ were performed in technical duplicates performed on at least three biological repeats (up to five times). *P* values were calculated using ANOVA with Dunnett’s multiple-comparison test comparing the lines to the PkATP4^OR^ line.

b NA, not applicable (as this is the comparator line).

Upon addition of the ATP4 inhibitor cipargamin, we observed a dose-dependent increase in the [Na^+^]_i_ for all the parasite lines tested (a representative curve is shown in Fig. 3B), with improved signal-to-noise ratios at the higher drug concentration of 100 nM. To distinguish between the responses of different orthologue replacement lines, we calculated and compared the rates of increase in [Na^+^]_i_ from steady state for 10 min following the addition of 100 nM cipargamin to the parasites, as well as the maximum increase in [Na^+^]_i_ over that time. This period of time was selected as previous data showed Na^+^ accumulation continuing to increase linearly at 10 min until the curve begins to flatten after 60 min (18). This linear increase 10 min after drug exposure was observed also for all the lines tested in our assays. When we compared the rates of increase in the [Na^+^]_i_ after 10 min of cipargamin exposure, we observed a significant difference only between the PfATP4^OR^ line compared to the control PkATP4^OR^ line (*P* = 0.0002) (Table 2 and Fig. 3C). The rate of [Na^+^]_i_ increase for the PfATP4^OR^ line (203.2 μM/s) was over 5 times faster than that for the PkATP4^OR^ line (35.8 μM/s) (Table 2) in the first 10 min after exposure to the inhibitor. None of the other lines were significantly different from the PkATP4^OR^ line with respect to their rate of [Na^+^]_i_ increase within the first 10 min after cipargamin exposure (*P* ≥ 0.2618) (Table 2 and Fig. 3C). When comparing the magnitudes of the increase in [Na^+^]_i_ among the parental and orthologue replacement lines over the 10-min exposure to cipargamin, we observed increases from steady state of between 18 and 35 mM for most lines, and these values were not significantly different from those of the PkATP4^OR^ line (*P* ≥ 0.9972) (Table 2). However, exposure to 100 nM cipargamin in the PfATP4^OR^ line for 10 min produced a maximum increase in [Na^+^]_i_ of 138.6 mM (Table 2), significantly higher than that in the PkATP4^OR^ line (*P* = 0.0007) (Table 2). The PvATP4^OR^ line experienced an intermediate maximum increase in [Na^+^]_i_ of 81.1 mM after cipargamin exposure, but this value was not significantly different from that of the PkATP4^OR^ reference line (*P* = 0.1312) (Table 2). Thus, the magnitude of this rapid, cipargamin-induced inhibition of Na^+^ efflux was lower for all non-falciparum orthologue replacement lines than those of the PfATP4^OR^ line and suggests that intracellular drug has an immediate impact on ATP4 function in P. knowlesi, but this is much more profound for the P. falciparum ATP4 than for its orthologues from other human-infecting members of the genus.

### Effect of orthologue replacement on pH_i_ (pH_cyt_).

**FIG 4:**
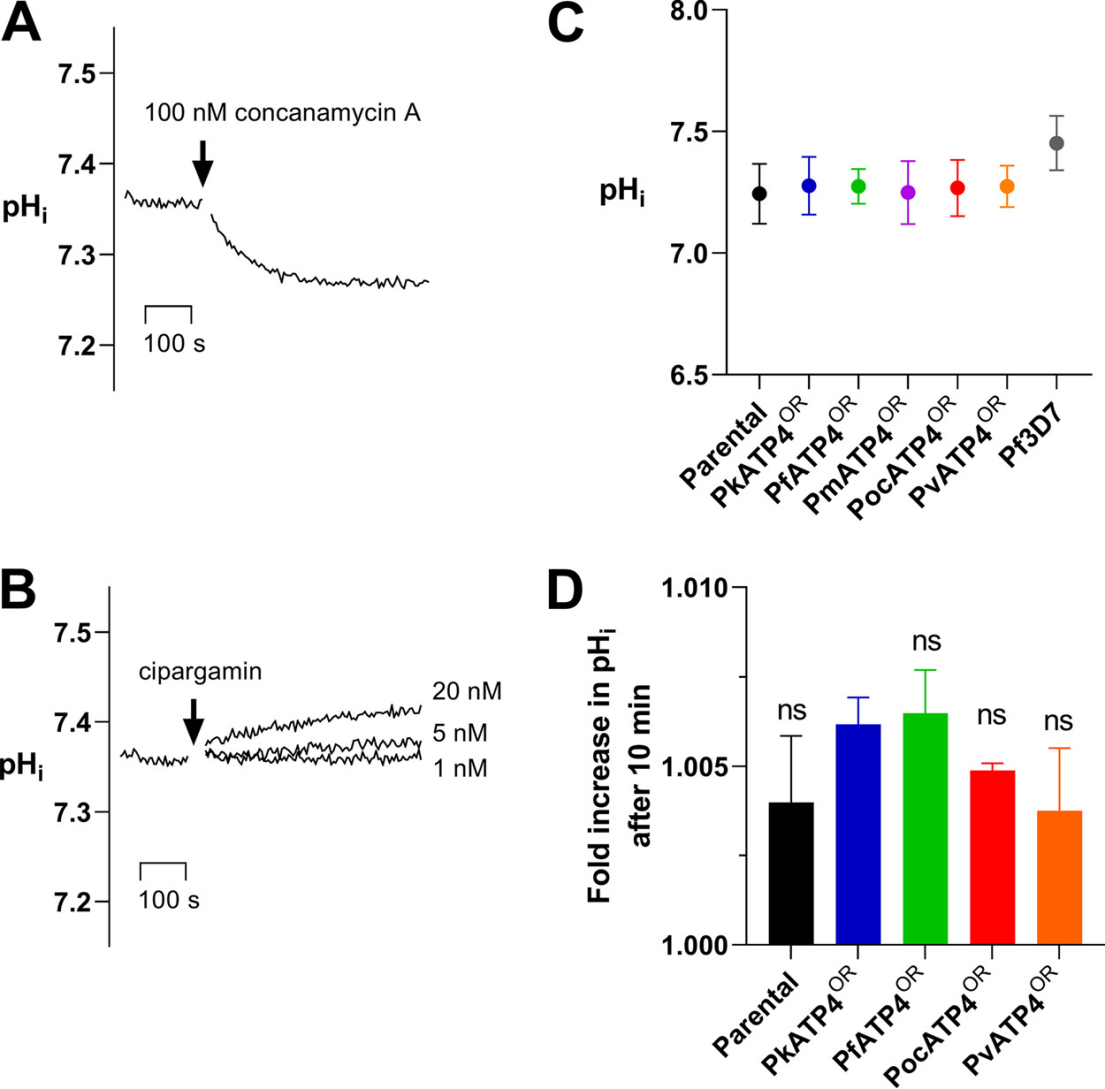
Resting cytosolic pH of orthologue replacement lines and the concentration-dependent increase in pH after addition of cipargamin. Trophozoite-stage parasites were loaded with the pH-sensitive fluorophore BCECF, and the fluorescence of the suspensions was measured in a spectrophotometer. (A) Addition of 100 nM concanamycin A, a V-type H^+^-ATPase inhibitor, caused a rapid acidification of the pH_i_ for the P. knowlesi parental line as shown before in P. falciparum (35). Shown here is a trace from a single experiment, but it is representative of results obtained on multiple occasions and in all lines. (B) Dose-dependent effect of adding the ATP4 inhibitor cipargamin on alkalinization of pH_i_ in the P. knowlesi parental line. Data shown are the average from at least 3 independent biological replicates ± SEM (C) Measurements of baseline pH_i_ show no significant difference among the P. knowlesi parental parasites and the orthologue replacement lines (pH_i_ ~7.25). The P. falciparum intracellular pH was slightly higher (7.45). (D) We determined the fold increase in pH_i_ after the addition of 100 nM cipargamin to the parasite lines. The fold increase was determined by dividing the pH_i_ at 10 min by the resting pH_i_ before the addition of cipargamin (*t* = 0 min). Data for the resting pH experiments are from technical duplicate experiments performed with at least five biological repeats (up to 10 times). Data from the fold change in pH experiments are from duplicate experiments performed on two to three separate occasions. ns, non significant (*P* > 0.05).

P. falciparum maintains a resting cytosolic pH (pH_cyt_) of around 7.3 through its plasma membrane-localized V-type H^+^-ATPase (35). Inhibition of this V-type H^+^-ATPase by the inhibitor bafilomycin A_1_ (35) or concanamycin A (36) induces a rapid acidification within the parasite cytosol. Using the pH-sensitive fluorophore BCECF [2′,7′-bis-(2-carboxyethyl)-5-(and-6)-carboxyfluorescein], we demonstrate that parental and transfectant P. knowlesi lines maintain a similar resting pH to that of P. falciparum,between 7.24 and 7.28 (Table S2). We show also that, in P. knowlesi, the addition of concanamycin A (100 nM) causes a rapid acidification of the cytosolic pH (Fig. 4A), similar to that observed for P. falciparum previously.

10.1128/mbio.01178-22.4 TABLE S2 Comparison of the resting pH_i_ and the fold increase in the pH_i_ after a 10-min exposure to 100 nM cipargamin in the P. knowlesi A1-H.1 parental and orthologue replacement lines. Download Table S2, DOCX file, 0.01 MB. Copyright © 2022 Mohring et al. 2022 Mohring et al. https://creativecommons.org/licenses/by/4.0/ This content is distributed under the terms of the Creative Commons Attribution 4.0 International license.

In contrast to inhibitors of the V-type H^+^-ATPase, which acidify of the cytosol, inhibitors of ATP4, a P-type ATPase, have been shown to rapidly alkalinize the cytosol in P. falciparum (17, 18). We demonstrate here that, in P. knowlesi, addition of cipargamin causes a dose-dependent increase in the cytosolic pH (Fig. 4B). In our experiments on the P. falciparum 3D7 line, we report an intracellular pH (pH_i_) of 7.45 ± 0.04, which was significantly different from that of the P. knowlesi PkATP4^OR^ line (*P* = 0.0291) (Table S2), suggesting resting pH is dependent on multifactorial differences in pH homeostasis control between the two parasite species and not governed by ATP4 specifically, as expected (35, 37). The orthologue replacement lines all displayed a resting pH_i_ similar to that of the parental P. knowlesi line (Fig. 4C).

In each line, a 10-min exposure to 100 nM cipargamin caused a small change compared to resting pH_i_ (Fig. 4D and Table S2). For the parental P. knowlesi line this was a 0.4% ± 0.1% increase in pH (Table S2), and it was almost identical to that shown by the orthologue replacement lines PkATP4^OR^ (0.6% ± 0.04% increase) and PfATP4^OR^ (0.6% ± 0.07% increase). The PocATP4^OR^ and PvATP4^OR^ lines were also tested and gave similar, small increases in pH (0.5% ± 0.01% and 0.4% ± 0.01%).

### Molecular modeling.

**FIG 5:**
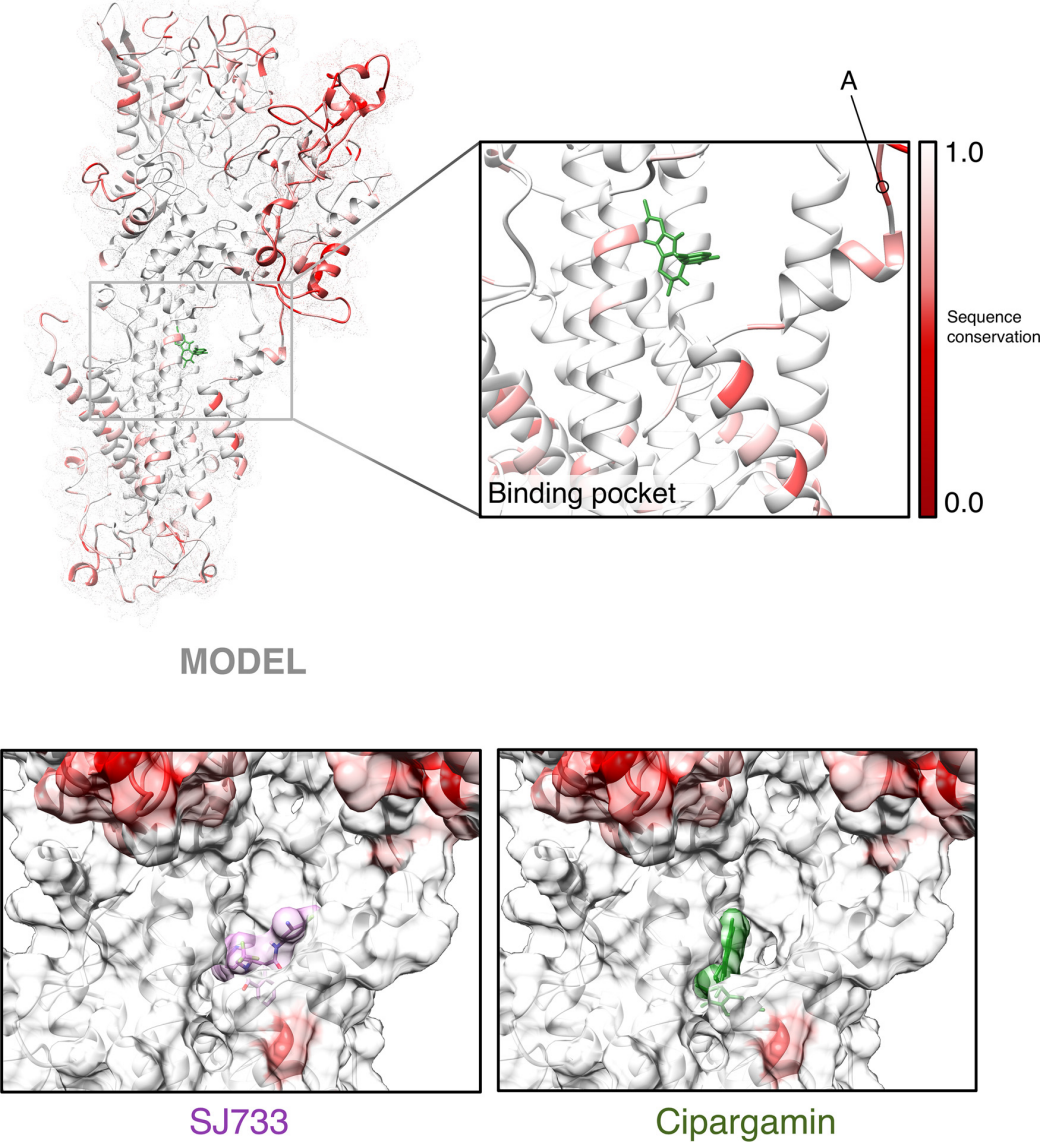
Molecular models of ATP4 orthologues and reidentification of the solvent-exposed spiroindolone drug-binding pocket. (Top panel) Structural model of PfATP4 colored by sequence conservation as determined by multiple-sequence alignment of *Plasmodium* ATP4 orthologues described in this study. Red indicates no sequence identity, whereas white indicates perfect conservation across all orthologues on a per-amino-acid basis. The inset shows the drug-binding pocket identified by docking drug molecules across the entire solvent-exposed protein surface. “A” indicates the 5-amino-acid peptide identified by Jimenez-Diaz et al. (18) as associated with SJ733 susceptibility. (Bottom panels) Surface maps with false coloring as described above showing SJ733 and cipargamin docked within the identified drug-binding pocket in the context of our PfATP4 model.

Alignment of each of the ATP4 orthologues from P. berghei and the human-infective species demonstrates that the differences in susceptibility do not appear to correlate with previous markers of PfATP4 resistance (Fig. S1). Indeed, three PfATP4 resistance-conferring residues identified through selection of P. falciparum with ATP4 inhibitors are conserved in the wild-type (WT) *atp4* gene sequence of all orthologues (18). Differences in orthologue susceptibility are thus likely to result from distinct mechanisms from those observed for *in vitro-*selected resistance. Previous work in P. berghei has also suggested that a 5-amino-acid loop of primary sequence diversity could also account for differences in species susceptibility (Fig. 5 and Fig. S1) (18). However, this sequence is more conserved among the human-infective species, particularly *P. ovale*, which harbors a single nonidentical amino acid and single similar amino acid within the loop.

10.1128/mbio.01178-22.1 FIG S1 Clustal alignment of full-length ATP4 amino acid sequences from 7 *Plasmodium* species. Red arrows indicate a selection of those residues reported in the literature to acquire nonsynonymous mutations in P. falciparum selection experiments with inhibitors cipargamin or SJ733 *in vitro*. The blue bracket indicates the “rodent loop” identified by Jiménez-Díaz et al. (18). Download FIG S1, PDF file, 0.2 MB. Copyright © 2022 Mohring et al. 2022 Mohring et al. https://creativecommons.org/licenses/by/4.0/ This content is distributed under the terms of the Creative Commons Attribution 4.0 International license.

In order to investigate structural differences among the protein orthologues that could explain our observations of phenotypic differences in ATP4 inhibitor susceptibility, we performed molecular modeling and docking experiments *in silico*. Models for PfATP4 were generated *de novo* using the Iterative Threading Assembler (I-TASSER) as described and compared with the primary model described by Jimenez-Diaz et al. (18), with good alignment noted (root mean square deviation [RMSD] of 2.4 Å over all atoms). Subsequently, threading models were built for the other ATP4 homologs based on this template, and structure-sequence similarity was compared by alignment (Fig. 5, upper panel). Overlay of the model ATP4 homolog structures revealed high similarity between the core transmembrane (TM) domains, with most structural dissimilarity occurring in the extracellular domain. Consistently, most sequence diversity, with respect to *Plasmodium* versus *Plasmodium* and *Plasmodium* versus non-*Plasmodium* ATP4 orthologues, also occurs in this extracellular domain (Fig. 5, upper panel; Clustal alignment in Fig. S1).

**FIG 6:**
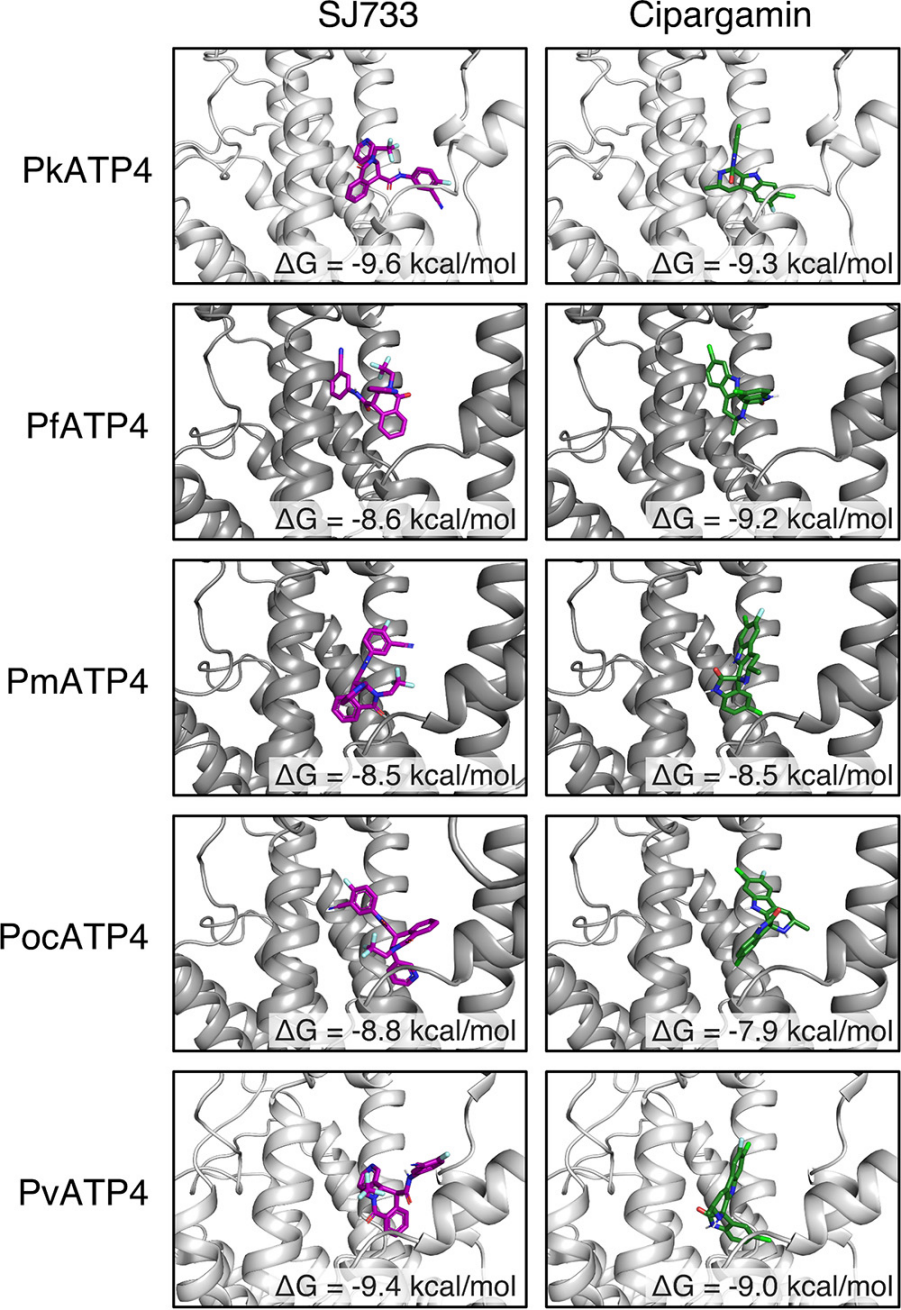
Structural models of top solutions of SJ733 and cipargamin docked to ATP4 orthologues. Solution structures of SJ733 (purple molecules, left column) and cipargamin (green molecules, right column) docked to model structures of ATP4 orthologues produced by AutoDock Vina. The inset label refers to the AutoDock-calculated free energy (Δ*G*) dissipated during formation of each protein-drug complex in kilocalories per mole. |ΔΔG| > 1 kcal/mol indicates a significant perturbation in binding.

Next, we docked the structures of (+)-SJ733 and cipargamin to each homolog using AutoDock, where the drug molecules were permitted to flex and rotate as chemistry and steric restrictions allowed. We reidentified the putative solvent-exposed drug-binding pocket noted by Jimenez-Diaz et al. (18), further supporting the validity of our models. Interestingly, the docking solutions for each homolog are structurally different, with the drug molecules rotating or lifting within the pockets to optimize stabilizing interactions (Fig. 6). This is likely due to slight differences in the diameter of the binding pocket based on the positions of the large transmembrane helices, which may or may not be artifactual to the modeling process. We calculated and compared the dissipated free energy of the top docking solution for cipargamin and SJ733 for each orthologue (Fig. 6). While PocATP4-cipargamin did exhibit a |ΔΔ*G*| of 1.3 kcal/mol with respect to PfATP4-cipargamin, predicting a significant lower stability of binding that may contribute to lower susceptibility of PocATP4^OR^ to cipargamin *in vitro*, there was no relationship between orthologues rank ordered by ΔΔ*G* (versus PfATP4) or fold difference in EC_50_ (versus PfATP4^OR^) for either compound. We note the short, 5-amino-acid loop of primary sequence diversity noted by Jimenez-Diaz that is structurally located near the drug-binding pocket (Fig. 5, upper right corner of inset, and Fig. S1). In all of the homology models, this loop does not have predicted secondary structure and occupies approximately the same spatial location, more than 20 Å away from the nearest atom of (+)-SJ733 or cipargamin. Furthermore, the helices located immediately downstream of this loop that are predicted to contact the drug molecules align well, suggesting no gross deformations in the drug pocket because of this diversity. While we cannot rule out that this loop may displace structures required for drug binding, our docking simulations did not identify any significant structural effects contributed by this loop.

We also compared our PfATP4 homology model to the DeepMind AlphaFold (AF)-predicted PfATP4 model (38), PfATP4_AF(120–1263), and noted that most of the structural disagreement lay in the N-terminal extracellular domain, with AF predicting greater secondary structure than the I-TASSER model. Around the drug-binding pocket examined above, AF predicts a smaller and more open binding pocket due to the α_5_ and α_6_ helices, which define the front of the pocket, being pushed downwards by ~6 Å. pLDDT confidence scores in the pocket range from 60 to 89 (low to confident) (Fig. S2). We used the AF structure as a threading template to resolve a new model structure, PocATP4_AF. PocATP4 was selected because the PfATP4^OR^ and PocATP4^OR^ lines consistently defined the maximum and minimum EC_50_ values to all three ATP4-targeting drugs. We noted similar alignment and structural features with these AF models as with the I-TASSER models, with no obvious features in the drug-binding pocket that would explain our *in vitro* drug susceptibility results. Dissipated free energies of docking were similar for SJ733 and cipargamin between the orthologues, consistent with no clear concordance between docking energies and the observed *in vitro* drug susceptibilities above.

10.1128/mbio.01178-22.2 FIG S2 Comparison of I-TASSER and AlphaFold models of PfATP4. (Left) Global structural alignment of PfATP4 models produced by AlphaFold (blue) and ITASSER (tan) with the inset window displaying the drug-binding pocket. An arrow indicates the primary structural perturbation in the drug-binding pocket, a displacement of helices a_5_ and a_6_ downwards by ~6Å in the AlphaFold model compared to the I-TASSER models. (Right) AlphaFold confidence map colored by pLDDT score (exported from the AlphaFold structural repository with UniProt identifier A0A143ZZK9). Download FIG S2, PDF file, 0.3 MB. Copyright © 2022 Mohring et al. 2022 Mohring et al. https://creativecommons.org/licenses/by/4.0/ This content is distributed under the terms of the Creative Commons Attribution 4.0 International license.

## DISCUSSION

In this study, we successfully generated a series of transgenic P. knowlesi orthologue replacement (OR) lines *in vitro* in which the endogenous *pkatp4* locus was replaced by a recodonized *pkatp4* coding region or the orthologous coding region from P. falciparum, *P. malariae*, *P. ovale* subsp. *curtisi*, or P. vivax. Each orthologue replacement transgenic line displayed a similar growth pattern to the parental P. knowlesi line, and susceptibility data were generated for each antimalarial compound of interest. We found evidence of significant orthologue-specific differences in parasite susceptibility to three chemically unrelated ATP4 inhibitors, but not to comparator drugs, among the P. knowlesi OR lines. The PfATP4^OR^ transgenic line of P. knowlesi was significantly more susceptible than the PkATP4^OR^ comparator to cipargamin, PA21A092, and SJ733. In contrast, the PocATP4^OR^ transgenic line was significantly less susceptible to these ATP4 inhibitors than the comparator line. The PmATP4^OR^ and PvATP4^OR^ lines were, however, not significantly different from the control PkATP4^OR^ line for two of the three ATP4 inhibitors (Table 1). Despite variation in the resting intracellular sodium concentrations among P. knowlesi lines expressing each of the five orthologues, we were able to measure significant differences in the magnitude of rapid, cipargamin-induced Na^+^ influx and the maximum increase in [Na^+^]_i_, between the P. falciparum OR line and each of the other four lines (Table 2), suggesting a real difference in the immediate functional impact of drug exposure on ATP4. In parallel experiments, cipargamin-induced increase in intracellular pH was observed in all lines, but was not significantly affected by the ATP orthologue present. Taken together, our findings confirm that species-specific susceptibility differences previously observed in rodent malaria and in *ex vivo* studies of human isolates are partly or wholly enshrined in the primary amino acid sequences of the respective ATP4 orthologues (18, 25). Our findings also confirm P. knowlesi as an important *in vitro* model for studying drug susceptibility in non-falciparum malaria parasites (24, 34), adding to its recently demonstrated utility for vaccine studies in P. vivax (26, 27, 39–42).

In other studies of the susceptibility of human malaria parasites to ATP4 inhibitors, cipargamin was shown to be effective against P. falciparum and P. vivax in *ex vivo* and *in vivo* studies, whereas the pyrazoleamide PA21A092 was more potent against P. vivax (15, 19, 43). However, in our *in vitro* studies, we showed that P. falciparum 3D7 was over 6-fold more susceptible than P. knowlesi A1-H.1 to the ATP4 inhibitors cipargamin, PA21A092, and SJ733 when tested under identical conditions (24). Furthermore, our recent *ex vivo* drug screens with cipargamin also performed under identical conditions confirmed that P. falciparum samples were more susceptible than those of *P*. *malariae* and *P*. *ovale* samples (25). The experiments enabled by the orthologue replacement lines have generated EC_50_ estimates suggestive of three distinct levels of enzymatic susceptibility (EC_50_ of PfATP4^OR^ ≪ PmATP4^OR^ ≈ PkATP4^OR^ ≈ PvATP4 ≪ PocATP4^OR^) and indicate that this appears to be universal for distinct ATP4 inhibitor classes. Future work to extend the panel of ATP4 inhibitors will enable us to examine whether this rank order of susceptibility remains the same or varies for distinct inhibitor structures.

Cipargamin causes a rapid influx in Na^+^, which followed a species-specific pattern, with a clear dichotomy in the magnitude of [Na^+^]_i_ flux between the PfATP4^OR^ line and the other P. knowlesi OR transgenic lines. This suggests a particularly profound functional impact of drug on PfATP4. The use of a single high cipargamin concentration may not be ideal to compare species differences. It may be preferable in future to use a preset multiple of the EC_50_ value (e.g., 5× the EC_50_ value) considering the wide variability of drug susceptibilities. For example, 100 nM cipargamin represents 60× the PfATP4^OR^ EC_50_ value but only around 3× the PocATP4^OR^ EC_50_ value. Alternatively, full dose-response curves for disruption of [Na^+^]_i_ regulation could be generated, as described for P. falciparum previously (17). Our understanding of these P-type ATPases remains inadequate, as well as how these interact with other cellular processes to regulate intracellular cation concentrations and pH.

Our data represent an important step forward in our understanding of ATP4 inhibitors. Recent phylogenetic analyses place PfATP4 into a distinct subgroup of P-type ATPases called “ATP4-type ATPases” (44). These are restricted to apicomplexan parasites (including *Toxoplasma* and *Plasmodium*) and closely related organisms, such as chromerids and dinoflagellates, and are absent in other eukaryotes (44). Mutations in PfATP4 are associated with resistance to a wide range of novel antimalarial candidates, including spiroindolones, MMV Malaria Box compounds, pyrazoles, and dihydroisoquinolones (15, 18, 19, 21), most of which show minimal toxicity against mammalian cells. The *in silico* modeling relied on a previously determined crystal structure from a rabbit calcium pump and would therefore benefit in the future from availability of the empirically derived structure of an ATP4-type ATPase from another apicomplexan or closely related organism. Jimenez-Diaz et al. previously used *in silico* modeling based on the rabbit calcium pump to explain species differences in susceptibility to SJ733 between the murine malarias and P. falciparum (18). In that study an important 5-amino-acid variant sequence loop was identified near the binding site that may explain the variations in SJ733 susceptibility between species (18). Most of the structural and sequence variations between the ATP4 orthologues were found in the extracellular domain. We could not identify bulkier amino acid side chains in the drug-binding pocket that might be responsible for steric constraints underlying the species differences in susceptibility to cipargamin and SJ733. Dissipated free energies of protein-drug docking did not reveal an orthologue rank order consistent with our *in vitro* observations. The 5-amino-acid sequence identified previously is spatially distant from the nearest atom of SJ733 and cipargamin and does not have the predicted secondary structure. We have been able to supplement this work with the use of AlphaFold structural predictions, and these provided results consistent with the threaded I-TASSER model. From our models, it is not obvious how this short loop mediates the significant differences in drug sensitivity observed for each orthologue, but it could be that species-specific differences in loop rigidity moves the conserved α_5_ and α_6_ into stabilizing or destabilizing interactions. Critically, the orthologous loop that is most conserved with the sequence in PfATP4 is found in *P. ovale*—the least sensitive ATP4 orthologue. Further work is required to pinpoint key sequence differences responsible for these drug susceptibility differences.

Our findings argue for the exploration of drug efficacy in human clinical trials with arms exposed to multiple-species malaria. The observed differences may reflect only a marginal impact on clinical efficacy for cipargamin: for example, where an EC_50_ estimate of 40 nM for the PocATP4 orthologue indicates that the drug retains excellent potency, but may mean certain species more easily acquire resistance or may require different dosage regimens. Conversely, it is unlikely that infections with either *P. ovale* subsp. *curtisi* or *P. ovale* subsp. *wallikeri* (these two species are very close relatives, with minimal divergence of “housekeeping” enzyme sequences) would respond well to treatment with either PA21A092 or SJ733, a finding with relevance for malaria treatment in sub-Saharan Africa, where these two species are widely distributed (45). Notably, none of the ATP4 orthologues appeared to result in any reduction in parasite fitness. Previous studies of ATP4 resistance mutations in P. falciparum reported variable effects on parasite growth, with some mutations resulting in a fitness cost (18), while other mutations produced no fitness cost (46). In our study, we compared growth of individual lines across only one life cycle duration. Other parasite fitness tests involve pairing wild-type with mutant lines and assessing growth across multiple life cycles (e.g., 20 to 30 days in reference 18). This approach, combining two lines in one culture at the same inoculum and comparing their growth across several growth cycles, may reveal differences in the fitness of different ATP4 orthologues compared to others.

In this study, we utilized the P. knowlesi model to explore species differences in ATP4 inhibitor susceptibility that we had identified in previous *in vitro* and *ex vivo* drug susceptibility studies (24, 25). This model has also been used to examine putative P. vivax drug resistance genes (42) and to study P. vivax malaria vaccine targets (41) and invasion (27). An advantage of using the P. knowlesi transfection model over the P. falciparum model is that P. knowlesi is phylogenetically more closely related to all the human malaria species (particularly P. vivax) than P. falciparum (33). Also, P. knowlesi possesses a more balanced genome AT content (62.5%) than P. falciparum and demonstrates transfection efficiency orders of magnitude higher (26, 39, 40). Our genome editing approach also has the key advantage of placing the orthologue gene into the ATP4 locus, directly under the endogenous P. knowlesi 5′ and 3′ untranslated regions (UTRs), providing significant advantages over exogenous episomal constructs or those controlled by generic expression cassettes. Although not used here, the lines generated are also markerless, so can be modified iteratively in future work. Our current analysis used orthologue sequences taken from reference genomes of each of the human infective species—but we still do not know how natural diversity in ATP4 from different species may affect ATP4 inhibitor susceptibility. The role of ATP4 variation identified from population genetics studies in each of the human infective species can be readily tested using this approach. Similarly, whether orthologue susceptibility and selected P. falciparum resistance mutations can interact to further modulate susceptibility in non-falciparum species could be investigated in this way. A limitation of our study is that we did not measure ATP4 orthologue expression or cellular localization in our transfectant lines. This would require antibodies to each orthologue or an appropriate tag, which was beyond the scope of the current study. However, given that ATP4 is essential and the orthologue replacements resulted in normal parasite growth rates, any difference in expression levels in the recodonized ATP4 sequences is likely small. Furthermore, the control recodonization of PkATP4 maintained parental inhibitor susceptibility and the orthologue replacement transgenics recapitulated the species differences observed for the susceptibility of *ex vivo* malaria parasites to cipargamin published by us previously (25).

Our study firmly establishes the existence of species-specific differences in susceptibility of human-infecting *Plasmodium* spp. to ATP4 inhibitors and provides compelling evidence that these differences lie within the primary sequence of the ATP4 protein orthologue in each parasite. We have also shown the enormous potential of P. knowlesi as a genetically tractable *in vitro* experimental workhorse for drug susceptibility studies of relevance to all the malaria parasites of humans.

## MATERIALS AND METHODS

### Parasite maintenance, transfection, and dilution cloning.

Human blood from all blood groups was obtained from the United Kingdom National Blood Transfusion Service. P. knowlesi is unable to grow in Duffy-negative (Fy−) red blood cells (RBCs) (40). Therefore, each new batch of red blood cells was tested first to assess whether they can support the parental P. knowlesi line at a normal 3- to 4-fold growth rate before being used to grow the transfectant lines. Parasites were maintained in complete medium, comprising RPMI 1640 (Invitrogen) with the following additions: 2.0 g/L sodium bicarbonate, 4.0 g/L d-glucose, 25 mM HEPES, 0.05 g/L hypoxanthine, 5 g/L AlbuMAX II, 0.025 g/L gentamicin sulfate, 2 mM l-glutamine, and 10% (vol/vol) horse serum (Pan Biotech; P30-0702) as described previously (47). Parasites were synchronized by using gradient centrifugation with 55% Nycodenz (Progen; product 1002424) in RPMI to enrich schizonts, followed by a 2-h incubation with 4-[7-[(dimethylamino)methyl]-2-(4-fluorophenyl)imidazo[1,2-*a*]pyridin-3-yl]pyrimidin-2-amine (compound 2), which inhibits parasite egress (48).

Tightly synchronized mature schizonts were transfected as described previously using the Amaxa 4D electroporator (Lonza) and the P3 primary cell 4D Nucleofector X kit L (Lonza; product V4XP-3024) (40). Ten microliters of DNA, including 20 μg pCas9/sg_*PkATP4* plasmid and 40 μg repair template pDonor_*ATP4^OR^* (containing the recodonized orthologue PfATP4, PkATP4, PmATP4, PocATP4, or PvATP4), was used for transfections to generate ATP4 transgenic lines. After 24 h, and at daily intervals for 5 days, the medium was replaced with fresh medium containing 100 nM pyrimethamine (Sigma; product P7771). Parasite clones were obtained by limiting dilution. Parasites were diluted to 0.3 parasite/100 μL, and 100 μL of 2% hematocrit culture was transferred to 96 flat-bottom plates in culture medium containing 2 mM l-GlutaMAX (Gibco; product 35050). After 7 days, the plate was screened for plaques in an assay modified from P. falciparum (49). Plaque-positive cultures were transferred to 24-well plates containing 1 mL medium with 2% hematocrit and used for genotyping when parasites appeared in culture.

### Cloning of transfection plasmids.

For cloning of pCas9/sg_ATP4 plasmids, two P. knowlesi target-specific 20-bp guide sequences were chosen with the Protospacer software (http://www.protospacer.com/) (50) (GCAGAAGGCTTTGAGAGTTC and TGTTCGTAAGTTACCCGCTG), with off-target scores of 0.0103 and 0.0105. Subsequently, each guide was inserted into the BtgZI linearized pCas9/sg plasmid by In-Fusion cloning (TaKaRa) using forward oligonucleotide TTACAGTATATTATT(N20)GTTTTAGAGCTAGAA and reverse oligonucleotide TTCTAGCTCTAAAAC(N20)AATAATATACTGTAA as described before (26, 51).

For cloning the donor plasmid pDonor_ATP4, homology regions were amplified from Plasmodium knowlesi parental A1-H.1 genomic DNA by using CloneAmp polymerase (TaKaRa). The 5′ homology region was amplified with forward oligonucleotide atatgCCGCGGGTATACAAGAAGAAAATAGGCCTTTGAATAAG and reverse oligonucleotide atatgaACTAGTAACTTTTTAGGAAAACACGGAAAGTGGAAAAAAAG leading to a 719-bp fragment of PkATP4 5′ UTR. The 3′ homology region was amplified with forward oligonucleotide atatggaGCGGCCGCTGCAGAGGTGGAATAATCATCACTTGG and reverse oligonucleotide atatggaCCATGGGCGGTATACCCATACGGCGC generating a 748-bp region of the PkATP4 3′ UTR (lower case letters are random spacer bases and underlined indicate the restriction site sequences.). The homology regions were introduced into a plasmid backbone with SacII/SpeI and NotI/NcoI restriction sites. Sequences of SERCA-type Ca^2+^-transporting P-ATPase orthologues were retrieved from PlasmoDB (https://plasmodb.org): PkATP4 (PKNH_1312000), PfATP4 (Pf3D7_1211900), PmATP4 (PmUG01_13021900), PocATP4 (PocGH01_13021900), and PvATP4 (PvP01_1311100). Recodonized synthetic sequences representing each orthologue coding region were obtained from GeneArt and were designed to be flanked by SpeI and NotI restriction sites, permitting insertion between the homology regions following SpeI/NotI restriction digest. Each synthetic sequence was thus inserted into a plasmid, flanked with the 5′ and 3′ UTRs of *pkatp4* as homologous repair templates (pDonor_*pkatp4*). Together with a plasmid containing the Cas9 endonuclease, one of two guide RNAs and a positive/negative selection marker cassette, each pDonor plasmid was transfected into synchronous late stage schizonts of P. knowlesi A1-H.1 strain as previously described (22). After genotyping, parasite lines were cloned by limiting dilution, resulting in 50% to 100% of the clones with successful replacement.

Plasmids for transfection were prepared by Midi-preps (Qiagen) and ethanol precipitated. The DNA pellet was washed twice with 70% ethanol and resuspended in sterile Tris-EDTA (TE) buffer.

### DNA analysis.

Genomic DNA from transfected parasite lines was extracted (Qiagen) and analyzed by PCR with SapphireAmp Fast PCR master mix (TaKaRa; product RR530B) using the following conditions: 1 min at 94°C, then 34 cycles of 5 s at 98°C, 5 s at 62°C and 10 s/kb at 72°C. The diagnostic oligonucleotides for the WT PkATP4 locus are forward oligonucleotide CTTCTAAGGGGTCTAAGAGAGGTAG and reverse oligonucleotide GTGTGTGCACTGCTAGGTACGG. The forward oligonucleotides for integration are as follows: PfATP^OR^, CCCTGCTGAACCTGTTTCTGGAC; PkATP4^OR^ and PocATP4^OR^, CTCCACATGCATCCCCGGCA; PmATP^OR^, GAACACCACATGTCTGCTGTGGTG; and PvATP4^OR^, CATTCCTGGCCACATGCATCCCT The reverse oligonucleotide for all orthologues was GTGTGTGCACTGCTAGGTACGG. The oligonucleotides for independent locus PkMTIP are forward oligonucleotide CCCGGGGCGTTTTCGCGTATCTGCGCTTTTTC and reverse oligonucleotide CCTAGGGGACAATATATCCTCACAGAACAACTTG.

### Parasite multiplication rate assays.

In the flow cytometry-based parasite multiplication rate assay, the fold increase in parasitemia following one round of asexual growth is measured. Purified schizonts were set up in technical duplicate cultures with human RBCs, at a 2% hematocrit and ~0.5% parasitemia in 24-well plates. Parasitemia was measured with a flow cytometry (fluorescence-activated cell sorter [FACS])-based assay before and after incubation at 37°C in a gassed chamber for 24 h. Samples were stained for 30 min with SYBR green I (Thermo Fisher Scientific; product S7563) and counted with the Attune flow cytometer (Thermo Fisher Scientific).

### Growth inhibition assay.

The ATP4 inhibitors cipargamin, PA21A092, and SJ733, as well as the established antimalarials chloroquine and dihydroartemisinin, were supplied by the Medicines for Malaria Venture, Geneva, Switzerland. Chloroquine stocks were prepared in sterile distilled water, but all other stocks wer prepared in dimethyl sulfoxide (DMSO).

Drug susceptibility of P. knowlesi parental and transgenic parasite lines was assessed as described previously with parasites exposed to the serial dilutions of the drugs for one complete life cycle (27 h) (24).

Parasite viability was determined using the SYBR green I fluorescence method (52, 53) as described elsewhere (24). Fluorescence was read using either a Spectramax M3 or iD5 microplate reader (Molecular Devices) at the 520-nm wavelength after excitation at 490 nm.

### Parasite [Na^+^]_i_ and pH_i_ measurements.

For both [Na^+^]_i_ and pH_i_ measurements, mature stage trophozoite parasites (36 to 40 h postinvasion for P. falciparum, 20 to 23 h postinvasion for P. knowlesi) were first isolated from their erythrocytes through treatment with saponin (0.05% [wt/vol]) (35).

The pH-sensitive fluorescent dye BCECF [2′,7′-bis-(2-carboxyethyl)-5-(and-6)-carboxyfluorescein] (Biotium; product 51011) was used to measure the cytosolic pH of the P. falciparum 3D7 line and the P. knowlesi parental (A1-H.1) and transfectant lines. Saponin-isolated trophozoite parasites were loaded with the acetoxymethyl ester of BCECF as described before (35). Thereafter, the parasites were suspended in physiological saline (120 mM NaCl, 5 mM KCl, 25 mM HEPES, 20 mM d-glucose, and 1 mM MgCl_2_ [pH 7.1]) in the presence or absence of various concentrations of ATP4 inhibitors in a 96-well microtiter plate (200 μL of parasite suspension per well). After being loaded into a 96-well microtiter plate, the fluorescence was measured at a wavelength of 520 nm after excitation at both 440 nm and 490 nm using a Spectramax M3 microplate reader (Molecular Devices) in a temperature-controlled chamber at 37°C.

For each experiment, a pH calibration was performed. Briefly, the BCECF-loaded parasites were suspended in a high-K^+^ saline (130 mM KCl, 25 mM HEPES, 20 mM d-glucose, and 20 mM MgCl_2_) at pH values of 6.8, 7.1, and 7.8. To this was added 15 μM nigericin (Tocris Bioscience; product 4312) which sets the intracellular pH to that of the surrounding saline. A linear regression can then be plotted between the calibration pH values and the intracellular fluorescence ratios (490 nm/440 nm). Then, for the experimental samples, this linear equation is used to convert fluorescence into pH units.

Intracellular sodium ([Na^+^]_i_) was measured using the Na^+^-sensitive fluorescent dye SBFI (Thermo Fisher Scientific; product S1263). Saponin-isolated trophozoites were loaded with the acetoxymethyl ester of SBFI as described by others (17). Thereafter, SBFI-loaded parasites were resuspended in physiological saline (as described above) at 37°C in the presence or absence of the ATP4 inhibitors. Calibration curves were set up using SBFI-loaded parasites suspended in calibration buffers with [Na^+^] concentrations between 0 and 130 mM (pH 7.3) containing 110 mM Na^+^/K^+^ gluconate, 30 mM Na^+^/K^+^ Cl, 1.2 mM CaCl_2_, 0.6 mM MgCl_2_, and 10 mM HEPES and the ionophore gramicidin D (17).

The parasites were loaded into a 96-well microtiter plate, and the fluorescence was measured at a wavelength of 515 nm after successive excitation at 340 nm and 380 nm. Calibration was performed as described previously to compare the relationship between the 340-nm/380-nm ratio and intracellular sodium (54, 55). The rates of increase in [Na^+^]_i_ observed 10 min after the addition of 100 nM cipargamin were calculated by fitting a linear regression equation (*y* = *mx* + *b*) to the data. The slope (*m*) represents the rate of change, and *b* represents the intercept. The maximum change in [Na^+^]_i_ after the addition of cipargamin was calculated by subtracting the minimum value for [Na^+^]_i_ (i.e., immediately before the addition of cipargamin) from the highest [Na^+^]_i_ achieved within the first 10 min of cipargamin exposure.

### Molecular modeling and docking.

Molecular models of PfATP4 were constructed using the molecular threading algorithm I-TASSER (56). Briefly, the amino acid sequence was modeled *de novo* and separately threaded through the crystal structure of the Oryctolagus cuniculus calcium pump (PDB no. 2DQS), a transmembrane (TM) Ca^2+^ ATPase found to have similar predicted TM domain structure, a technique used previously by Jimenez-Diaz et al. (18). The resulting molecular models of PfATP4 were then validated for structural similarity by alignment with the Jimenez-Diaz model and subsequently used as a threading template for the remaining P. knowlesi, P. vivax, *P. malariae*, and *P. ovale curtisi* ATP4 sequences.

The molecular structures of SJ733 and cipargamin were then docked to these structures using AutoDock Vina 1.1.2 over the entire solvent-exposed surface area as well as within a docking grid (22.5 Å in each dimension) constrained to the SJ733 binding pocket identified by Jimenez-Diaz et al. (18). Docking SJ733 to PfATP4 over the entire protein surface area reidentified the binding pocket identified by Jimenez-Diaz et al., and thus the constrained docking approach was used for the other species to investigate a greater number of docking configurations for each ATP4 homolog. The intrinsic Vina scoring function (57) was used to calculate and compare the dissipated free energy of the top docking solution for each structure.

Visualizations of the molecular models and docked structures were prepared using Chimera (58) and PyMol (version 2.4.0) (Schrödinger).

### Statistical analysis.

Data from the orthologue replacement lines were compared using ordinary one-way analysis of variance (ANOVA) with Dunnett’s multiple-comparison test to determine differences of the orthologue replacement lines compared to the PkATP4^OR^ line.
