# Supplementary material for: Cation ATPase (ATP4) Orthologue Replacement in the Malaria Parasite Plasmodium knowlesi Reveals Species-Specific Responses to ATP4-Targeting Drugs
Source: mBio. 2022 Oct 3;13(5):e01178-22. doi: 10.1128/mbio.01178-22 (PMC9600963; doi:10.1128/mbio.01178-22)
Supplement: TABLE S1 [file mbio.01178-22-s0003.docx]

**Supplementary Table 1:** Fold Difference in EC_50_ values among the *P. knowlesi* parental and orthologue replacement lines.

| **Parasite line** | ***Fold difference in EC_50_ values compared with PfATP4^OR^ line** | | | | |
| --- | --- | --- | --- | --- | --- |
|  | **Cipargamin** | **PA21A092** | **SJ733** | **Chloroquine** | **Dihydroartemisinin** |
| **Parental** | 5.87 | 4.43 | 5.28 | 1.00 | 0.79 |
| **PkATP4^OR^** | 8.33 | 7.08 | 7.96 | 1.13 | 0.98 |
| **PfATP4^OR^** | 1.00 | 1.00 | 1.00 | 1.00 | 1.00 |
| **PmATP4^OR^** | 5.83 | 5.21 | 3.35 | 0.90 | 0.97 |
| **PocATP4^OR^** | 21.0 | 33.7 | 14.9 | 0.85 | 0.96 |
| **PvATP4^OR^** | 14.1 | 5.33 | 7.66 | 0.97 | 0.77 |
|  |  |  |  |  |  |
| **PkA1-H1** | 3.77 | 3.58 | 5.44 | 0.85 | 1.10 |
| **Pf3D7** | 0.55 | 0.57 | 0.91 | 0.46 | 2.32 |

* Fold difference was calculated by dividing the mean EC_50_ value for the parental *P*. *knowlesi*, *P*. *falciparum* or orthologue replacement lines with the mean PfATP4^OR^ EC_50_ value using data from Table 1.
