# Supplementary material for: Cation ATPase (ATP4) Orthologue Replacement in the Malaria Parasite Plasmodium knowlesi Reveals Species-Specific Responses to ATP4-Targeting Drugs
Source: mBio. 2022 Oct 3;13(5):e01178-22. doi: 10.1128/mbio.01178-22 (PMC9600963; doi:10.1128/mbio.01178-22)
Supplement: TABLE S2 [file mbio.01178-22-s0004.docx]

**Supplementary Table 2:** Comparison of the resting pH_i_ and the fold increase in the pH_i_ after a 10 minute exposure to 100 nM cipargamin in the *P. knowlesi* A1-H.1 parental and orthologue replacement lines.

| **Parasite line** | **Resting pH_i_**  **(p value)** | **Fold change in pH_i_ (10 min after cipargamin exposure) (p value)** |
| --- | --- | --- |
| **Parental** | 7.24 ± 0.04 (0.9834) | 1.004 ± 0.0010 (0.2343) |
| **PkATP4^OR^** | 7.28 ± 0.05 (n/a) | 1.006 ± 0.0004 (n/a) |
| **PfATP4^OR^** | 7.28 ± 0.03 (>0.9999) | 1.006 ± 0.0007 (0.9959) |
| **PmATP4^OR^** | 7.25 ± 0.06 (0.9963) | ND |
| **PocATP4^OR^** | 7.27 ± 0.05 (0.9998) | 1.005 ± 0.0001 (0.7092) |
| **PvATP4^OR^** | 7.28 ± 0.04 (>0.9999) | 1.004 ± 0.0010 (0.1732) |
|  |  |  |
| **Pf3D7** | 7.45 ± 0.04 (0.0291) | ND |

Resting pH_i_ was measured in BCECF-loaded saponin-isolated late stage trophozoites suspended in physiological saline.

All data show the mean ± the standard error of the mean. Data for the resting pH experiments are from technical duplicate experiments performed on at least five biological repeats (up to ten times). Data from the fold change experiments are from duplicate experiments performed on two to three separate occasions.

* p values were calculated using ANOVA with Dunnett’s multiple comparisons test comparing the lines to the PkATP4^OR^ line.

ND = Not Determined

n/a = not applicable (as this is the comparator line)
