# Supplementary figures and images for: Cation ATPase (ATP4) Orthologue Replacement in the Malaria Parasite Plasmodium knowlesi Reveals Species-Specific Responses to ATP4-Targeting Drugs
Source: mBio. 2022 Oct 3;13(5):e01178-22. doi: 10.1128/mbio.01178-22 (PMC9600963; doi:10.1128/mbio.01178-22)

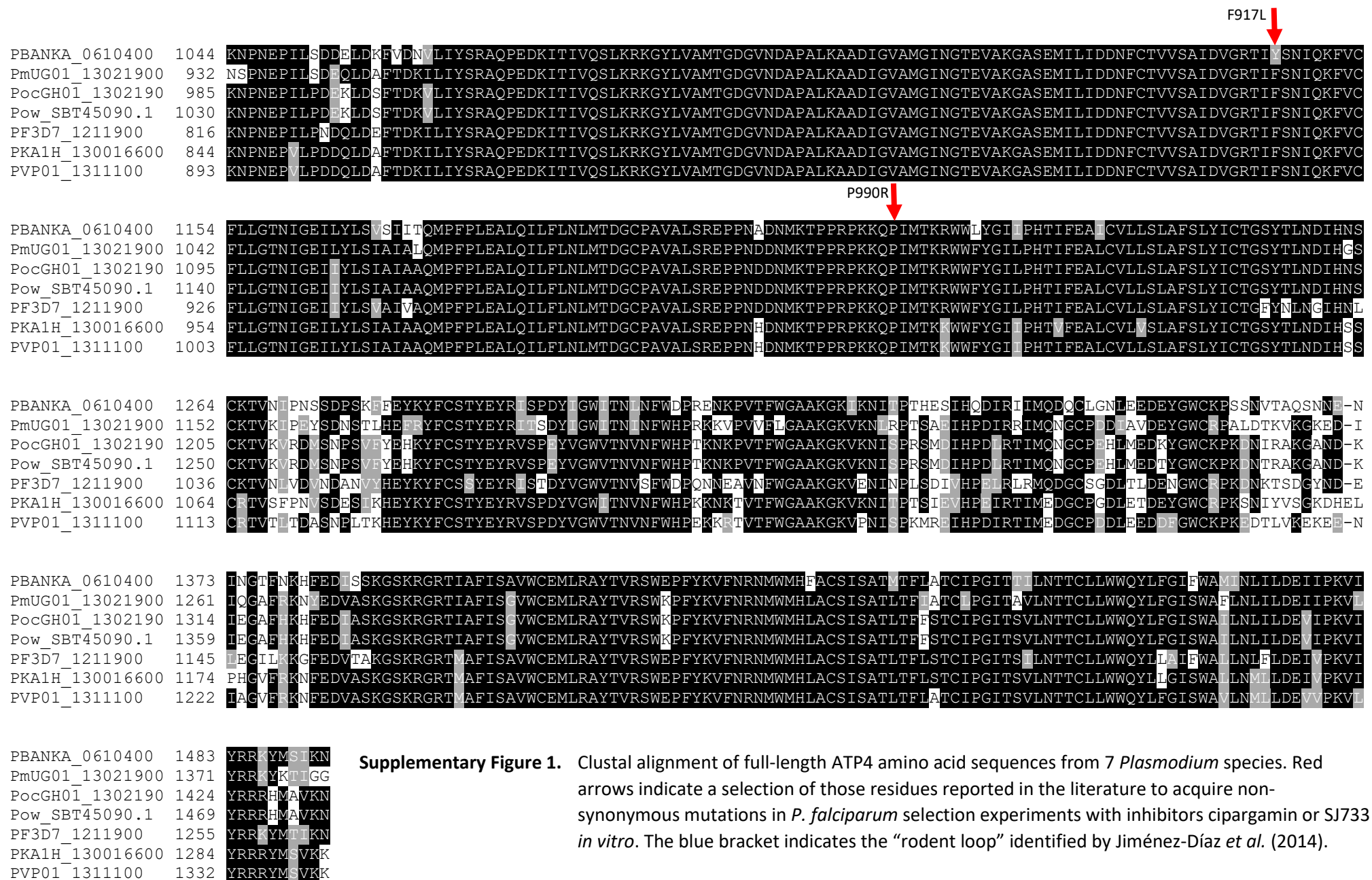

Supplement: FIG S1 [file mbio.01178-22-s0001.pdf]
