# Supplementary material for: Cation ATPase (ATP4) Orthologue Replacement in the Malaria Parasite Plasmodium knowlesi Reveals Species-Specific Responses to ATP4-Targeting Drugs
Source: mBio. 2022 Oct 3;13(5):e01178-22. doi: 10.1128/mbio.01178-22 (PMC9600963; doi:10.1128/mbio.01178-22)

Figure S2. Comparison of I-TASSER and AlphaFold models of PfATP4. Left: global structural alignment of PfATP4 models produced by AlphaFold (blue) and I-TASSER (tan) with inset window displaying the drug-binding pocket. Arrow indicates the primary structural perturbation in the drug binding pocket, a displacement of helices a_5 and a_6 downwards by approximately 6Å in the AlphaFold model compared to the I-TASSER models. Right: AlphaFold Confidence Map coloured by pLDDT score (source: exported from AlphaFold structural repository with UniProt identifier A0A143ZZK9).

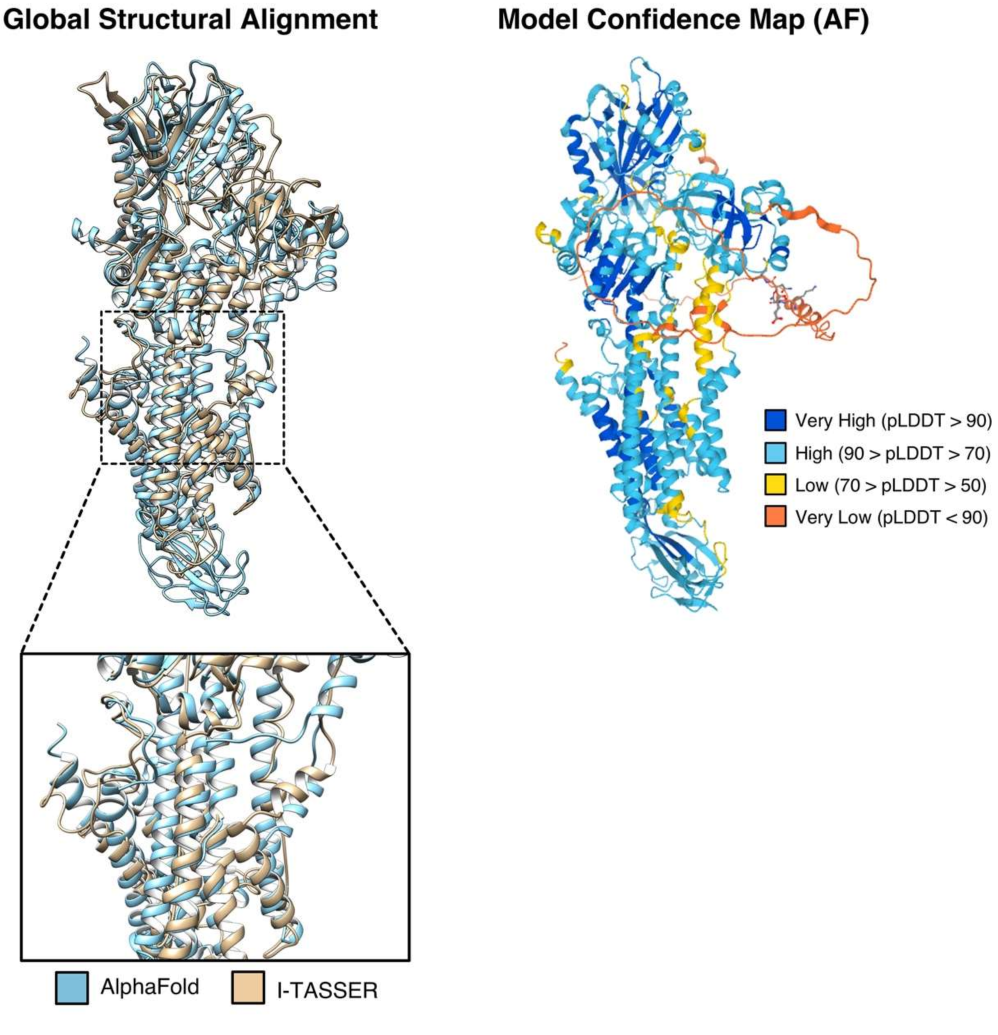

Supplement: FIG S2 [file mbio.01178-22-s0002.pdf]
